# Tree shrew (*Tupaia belangeri*) as a novel laboratory disease animal model

**DOI:** 10.24272/j.issn.2095-8137.2017.033

**Published:** 2017-05-18

**Authors:** Ji Xiao, Rong Liu, Ce-Shi Chen

**Affiliations:** ^1^Medical Faculty of Kunming University of Science and Technology, Kunming Yunnan 650500, China; ^2^Key Laboratory of Animal Models and Human Disease Mechanisms of the Chinese Academy of Sciences and Yunnan Province, Kunming Institute of Zoology, Chinese Academy of Sciences, Kunming Yunnan 650223, China

**Keywords:** Tree shrew (*Tupaia belangeri*), Animal model, Transgenic, Disease

## Abstract

The tree shrew (*Tupaia belangeri*) is a promising laboratory animal that possesses a closer genetic relationship to primates than to rodents. In addition, advantages such as small size, easy breeding, and rapid reproduction make the tree shrew an ideal subject for the study of human disease. Numerous tree shrew disease models have been generated in biological and medical studies in recent years. Here we summarize current tree shrew disease models, including models of infectious diseases, cancers, depressive disorders, drug addiction, myopia, metabolic diseases, and immune-related diseases. With the success of tree shrew transgenic technology, this species will be increasingly used in biological and medical studies in the future.

## INTRODUCTION

Animal models are used to simulate complex biological phenomena and human disease characteristics. Laboratory animal models are essential for addressing complicated biological problems, understanding the mechanisms of human diseases, and testing the efficacies of therapeutics. To date, *Drosophila*, zebrafish (*Brachydanio rerio*), frog (*Xenopus laevis*), mouse (*Mus musculus*), rat (*Rattus norvegicus*), rabbit (*Oryctolagus cuniculus*), dog (*Canis lupus familiaris*), pig (*Sus scrofa domesticus*), and rhesus macaque (*Macaca mulatta*) have been applied as animal models in the simulation of human cancers, as well as cardiovascular, neurological, metabolic, infectious, and autoimmune diseases (Buchholz, 2015; Jiang et al., 2011; Kazama, 2016; Xu, 2011; Xue et al., 2014). While mature animal models have propelled medical research considerably, no perfect model for human disease currently exists. Each animal model has its own unique advantages and disadvantages. Mice, e.g., have been widely applied due to their small size and clear genetic background; however, the significant genetic differences between rodents and humans mean that such models are unsatisfactory for certain diseases, such as hepatitis and AIDS (Glebe & Bremer, 2013). Compared with other experimental animals, non-human primates are genetically close to humans, allowing for the relatively accurate simulation of human pathology and physiology. However, their high cost and low reproductivity severely limit their use as experimental models. Therefore, the development of novel animal models to balance the advantages and disadvantages for specific disease research is critical.

The tree shrew (*Tupaia belangeri*) belongs to Mammalia, Scandentia, and Tupaiidae (Wang, 1987). It lives mainly in Southeast Asia and is characterized by short reproductive and life cycles, high reproductivity (4-6 months from birth to adulthood, with 2-6 offspring born each time), moderate size (adult weight 120-150 g), and easy feeding. The physiological features of the tree shrew, such as anatomy, neurodevelopment, and psychological stress, are highly similar to those of primates, including humans. For example, based on their mixed insectivorous and frugivorous diets, tree shrews are adept at climbing and moving with ease. Furthermore, tree shrews have a relatively well developed and arranged visual thalamus, resulting in acute visual and color discrimination abilities (Ranc et al., 2012). The primary visual cortex branch of the tree shrew is similar to that of primates, but not of rats, indicating that their visual system is closer to that of primates than that of rodents (Caspi et al., 2003; Veit et al., 2011, 2014; Wang et al., 2013b). The amygdaloid nucleus/hippocampus rate of tree shrews is much greater than that of rodents, allowing tree shrews to more easily accomplish memory tasks (Khani & Rainer, 2012; Nair et al., 2014; Wang et al., 2013b). Because of these advantages, a growing number of tree shrew disease models have been created (Xu et al., 2013). In 2012, the Kunming Institute of Zoology of the Chinese Academy of Sciences (KIZ, CAS) completed genome sequencing of the Chinese tree shrew, which showed that the nervous, immune, and metabolic systems of the tree shrew were very close to those of humans (Fan et al., 2013). The tree shrew database website (http://www.treeshrewdb.org/) was built based on the tree shrew genome sequence data (Fan et al., 2014). In addition, 3 482 tree shrew proteins have been predicted to be drug targets for cancer chemotherapy, depression, and cardiovascular diseases (Zhao et al., 2014). These studies have greatly facilitated the application of the Chinese tree shrew as an animal model in biomedical research. To date, 1 136 papers related to the tree shrew have been published.

## INFECTIOUS DISEASES MODELS

### Hepatitis virus infection models

Hepatitis is a serious health problem worldwide (Gravitz, 2011; Thun et al., 2010; Yan et al., 2012). However, it is difficult to identify suitable animal models for hepatitis research. In recent decades, studies have shown the tree shrew to be a practical small-animal model for experimental studies on the hepatitis virus, including hepatitis B, C, D, and E. Su (1987) and Pang et al. (1981) inoculated HBV into wild tree shrews and were able to detect the integrated HBV DNA, suggesting that HBV infection could be established in tree shrew models. Walter et al. (1996) also detected HBV DNA replication and viral protein expression in HBV-infected tree shrew liver. Experimental chronic hepatitis B infection in neonatal tree shrews further improved the efficiency of infection and more accurately simulated human chronic HBV infection (Liang et al., 2006; Wang et al., 2012a; Yang et al., 2009). Neonatal tree shrews can be persistently infected with HBV, and hepatic histopathological changes observed in chronically HBV-infected animals are similar to those observed in HBV-infected humans (Ruan et al., 2013). Additionally, chronic HBV infection combined with aflatoxin B1 has been shown to induce liver cancer in tree shrews (Li et al., 1999b; Yang et al., 2015). Furthermore, tumor necrosis factor α (TNFα) can inhibit hepatitis B virus replication in *Tupaia* hepatocytes (Xu et al., 2011). Sodium taurocholate co-transporting polypeptide (NTCP) has also been identified as a functional receptor for HBV and HDV based on a tree shrew model (Yan et al., 2012; Zhong et al., 2013).

Hepatitis C virus (HCV) is responsible for 150-200 million chronic infections and more than 350 000 deaths worldwide every year (Gravitz, 2011). Although chimpanzees and monkeys are considered natural infection hosts (Li et al., 2011), HCV has also been shown in tree shrews, with infected animals exhibiting intermittent viremia, high levels of ALT during the acute phase of infection, chronic hepatitis, liver steatosis, cirrhotic nodules, and accompanying tumorigenesis in the late stage (Amako et al., 2010). Irradiation has also been shown to increase the proportion of HCV infection in tree shrews (Xie et al., 1998). In addition, HCV can infect the primary hepatocytes of tree shrews (Amako et al., 2010; Zhao et al., 2002), with HCV receptors CD81, scavenger receptor BI (SR-BI), claudin-1, and occludin found to be essential factors for HCV entry and infection cycle in the primary hepatocytes of *Tupaia belangeri* (Tong et al., 2011).

Previous research successfully established HDV/HBV double infection in adult tree shrews to study virus pathogenesis and treatment (Li et al., 1995). In addition, tree shrews have been found to be susceptible to HEV when intravenously injected with swine genotype 4 HEV, with HEV RNA subsequently detected in the feces, liver, spleen, kidneys, and bile of infected tree shrews (Yu et al., 2016).

As one of only a few hepatitis susceptible animals, tree shrews can be infected with almost all hepatitis viruses, and can exhibit symptoms similar to those observed in humans. This makes the tree shrew an ideal animal for hepatitis disease research.

### Bacterial infection models

Pathogenic bacteria, such as *Staphylococcus aureus*, *Escherichia* *coli*, and *Pseudomonas aeruginosa*, can cause hundreds of thousands of deaths every year. Li et al. (2012) found that tree shrews are susceptible to *Staphylococcus aureus* and *Pseudomonas aeruginosa*. Zhan et al. (2014) infected Chinese tree shrews with *Mycobacterium tuberculosis* and found that infected animals developed serious symptoms similar to the clinical signs of active tuberculosis observed in humans. These studies indicate that tree shrews can be used to establish bacterial infection models.

### Other virus infection models

Other human viruses can also infect tree shrews. Herpes Simplex Virus type Ⅰ (HSV-1) and type 2 (HSV-2) can latently infect peripheral nervous system sensory neurons, and their reactivation can lead to recurring cold sores in tree shrews (Han et al., 2011). HSV-1 infection in the tree shrew trigeminal ganglion following ocular inoculation differs significantly from mice in the expression of key HSV-1 genes, including ICP0, ICP4, and latency-associated transcript (LAT), providing a valuable alternative model to study HSV-1 infection and pathogenesis (Han et al., 2011; Li et al., 2016a, b). As an important human pathogen of hand-foot-mouth disease (HFMD), Coxsackie virus A16 (CA16) has been successfully used to infect tree shrews to investigate its pathogenesis (Li et al., 2014). Foamy virus can naturally infect *Tupaia belangeri chinensis* and is highly related to simian foamy virus in *Macaca mulatta* (Huang et al., 2013a). Human H1N1 influenza-infected tree shrews have been shown to display mild to moderate systemic and respiratory symptoms and pathological changes in respiratory tracts (Yang et al., 2013). *Tupaia belangeri chinensis* has also been positively infected with enterovirus 71 (EV71) via oral administration, nasal dripping, and tail intravenous injection (Wang et al., 2012b).

### Immune-related disease models

Tree shrews can be used as an alternative to primates for studying immune-related diseases. The homology of the interleukin-2 (IL-2) protein sequence between tree shrews and humans is reportedly as high as 80% (Huang et al., 2013b). Although retinoic acid induction gene protein Ⅰ (RIG-I) has been lost in the Chinese tree shrew lineage, melanoma differentiation-associated protein 5 (MDA5) replaces its role in innate antiviral activity (Xu et al., 2016). In addition, chemokines in tree shrews might play similar roles as those in humans (Chen et al., 2014). Intraperitoneal injection of pristane and LPS (lipopolysaccharide) can induce systemic lupus erythematosus (SLE) (Ruan et al., 2016). Major histocompatibility complex (MHC) class Ⅰ and Ⅱ cell surface proteins are crucial for the self/non-self-discrimination of the adaptive immune system, with the MHC class Ⅰ gene recently characterized in the tree shrew successfully (Zhang et al., 2013).

## CANCER MODELS

### Hepatocellular carcinoma (HCC) models

Hepatocellular carcinoma is one of the most common malignancies worldwide (Raphael et al., 2012). Tree shrews can develop spontaneous HCC (Hofmann et al., 1981), but it can also be induced by aflatoxin B1 application (Reddy et al., 1976). The incidence of HCC has also been found to significantly increase when animals are infected with HBV and exposed to AFB1 (52.94%) compared with those solely infected with HBV (11.11%) or solely exposed to AFB1 (12.50%) (Yan et al., 1996). Alterations in *p53* and *p21* genes have also been detected in tree shrew HCCs induced by human HBV and/or AFB1 (Park et al., 2000; Su et al., 2003, 2004).

### Breast cancer models

Breast cancer is a common malignant tumor in women (Tong et al., 2014). Elliot et al. (1966) reported the first observed spontaneous breast cancer in tree shrew in the 1960s. Xia et al. (2012) further characterized a second case of spontaneous breast papillary tumor, with additional research showing that 50% of female tree shrews developed mammary tumors with long latency (about 7 months) following oral administration of carcinogens 7, 12-dimethylbenz(a)anthracene plus medroxyprogesterone acetate (DMBA and MPA). Interestingly, the *PTEN/PIK3CA* genes, but not the *TP53* gene, were frequently mutated in both the spontaneous and DMBA plus MPA-induced tree shrew mammary tumors (Xia et al., 2014; Xu et al., 2015). Overexpression of the *PyMT*oncogene by lentivirus can also efficiently induce mammary tumors in tree shrews within a month (Ge et al., 2016). Furthermore, KLF5, an important transcription factor in breast cancer initiation and development, is reported to be highly conserved between tree shrews and humans (Shao et al., 2017).

### Lung cancer models

Pulmonary cancer has the highest incidence and mortality in the world (Torre et al., 2016). The first spontaneous lung tumor in the tree shrew was reported in 1996 (Brack et al., 1996). The administration of DHPN (2, 2'-dihydroxy-di-n-propylnitrosamine) at a weekly dose of 250 mg/kg body weight subcutaneous for 80 weeks resulted in 78%-89% of animals developing pulmonary adenomas between 65 and 102 weeks (Rao & Reddy, 1980). In 2013, we administered a single dose of 4-(methylnitrosamino)-1-(3-pyridyl)-1-butanone (NNK, 100 mg/kg) via intraperitoneal injection to induce lung cancer in tree shrews, but did not find any lesions in the lungs after one year (unpublished data). Furthermore, PM10 (particulate matter with diameters of 10 μm or less) from Xuanwei bituminous coal dust was found to induce bronchial epithelial hyperplasia in tree shrews, although animals died within a week of prefusion (Chen et al., 2015). Most recently, tree shrews receiving iodized oil suspensions of 3-methylcholanthrene (3-MC) and diethylnitrosamine (DEN) via endotracheal instillation for 11 weeks developed bronchial epithelial atypical hyperplasia and carcinoma *in situ*, although all experimental animals died (Ye et al., 2016).

### Other tumor models

Spontaneous lymphoma was first observed in tree shrews in 1996 (Brack et al., 1996). Overexpression of H-Ras (human) and shP53 (tree shrew) by lentivirus for 139 days was shown to induce glioma tumors in tree shrews (Tong et al., 2017). Compared with corresponding mouse gliomas, tree shrew gliomas are more similar to human glioblastomas in terms of gene expression profile (Tong et al., 2017).

## METABOLIC DISEASE MODELS

### Diabetes models

Diabetes along with hyperglycemia is a metabolic disease. There are about 285 million diabetic patients worldwide (Shaw et al., 2010). Rabb et al. (1966) reported that spontaneous diabetes with the same ketosis, alopecia, and cataract phenotypes as observed in human diabetes was also found in the Philippine tree shrews (*Urogale everetti)*. Several research groups have successfully induced type 1 diabetes in tree shrews using streptozotocin (STZ) (Ishiko et al., 1997; Xian et al., 2000), which is widely used to establish diabetic animal models by selectively killing β cells (Srinivasan & Ramarao, 2007). Li et al. (2010) used a high-glucose-fat-diet in combination with dexamethasone to induce type 2 diabetes in tree shrews (Li et al., 2010). Wu et al. (2013) also established similar type 2 diabetes in tree shrews using different doses of STZ (60, 70, and 80 mg/kg). Furthermore, a diabetic nephropathy tree shrew model was successfully induced by a high-sugar and high-fat diet and four injections of STZ, with bone-marrow mesenchymal stem cell transplantation also demonstrated to improve insulin resistance (Pan et al., 2014).

### Fatty liver disease models

With economic development and an increase in the standard of living, fatty liver disease has become increasingly common (Qian et al., 2007). Tree shrews treated with alcohol solutions (10% and 20%) for two weeks were found to develop fatty liver-like pathological changes and alterations (Xing et al., 2015), while Meng et al. (2003) found that Rosiglitazone could prevent fatty liver development in the tree shrew. Using a high fat, cholesterol, and cholate diet, Zhang et al.(2015, 2016) successfully established non-alcoholic non-obese fatty liver disease (NAFLD) in tree shrews; interestingly, the inhibition of lipoprotein lipase by Poloxamer 407 improved the severity of steatosis and reduced inflammation (Zhang et al., 2015). Additionally, 20% sucrose and 1% cholesterol added to their standard diet resulted in massive gallstones in the tree shrew (Schwaier, 1979). Compared with the mouse fatty liver disease model, the tree shrew model shows a shorter onset time and more obvious symptoms (Zhang et al., 2015).

### Blood vascular disease models

Tree shrews are regarded as an animal model resistant to atherosclerosis. Liu et al. (2010) showed that tree shrews are resistant to atherosclerosis because of low cholesteryl ester transfer protein expression and low phospholipid transfer protein activities. Li et al. (1999a) induced thrombotic cerebral ischemia in tree shrews using a photochemical approach, with Feng et al. (2011) showing that cerebral ischemia caused a predominant increase in TLR4 protein expression in the tree shrew hippocampus. A blood stasis syndrome model was also successfully established in tree shrews with high-dose carrageen glue (Zhang et al., 2016)

## MENTAL DISEASE MODELS

### Depression models

Depression, a common mental disease, influences about 20% of people worldwide (Nestler et al., 2002). Tree shrews are diurnal animals, which can be advantageous in certain research compared with using nocturnal animals such as rodents. Previous studies have shown a variety of physiological changes, such as constantly elevated levels of urinary cortisol and norepinephrine and reduced body weight, in subordinate, but not dominant males (Fuchs et al., 1995; Magariñ os et al., 1996). Fuchs & Flügge (2002) described the physiology, brain function, and behaviors of subordinate tree shrews. Coexistence of two males with visual and olfactory contact led to a stable dominant/subordinate relationship, with the subordinate showing obvious changes in behavior, neuroendocrine, and central nervous activity similar to the symptoms observed in depression patients (Van Kampen et al., 2002). Clomipramine treatment has also been shown to counteract the behavioral and endocrine effects of chronic psychosocial stress in tree shrews (Fuchs, 2005; Fuchs et al., 1996), whereas diazepam has no beneficial effects on stress-induced behavioral and endocrine changes in male tree shrews (Van Kampen et al., 2000). Wang et al. (2011) examined two male tree shrews for one hour of direct conflict (fighting) and 23 hours of indirect influence (e.g., smell, visual cues) per day for 21 days and found that the subordinate tree shrews showed alterations in body weight, locomotion, avoidance behavior, and urinary cortisol levels in the final week of social conflict. Schmelting et al. (2014) reported that agomelatine normalized the core body temperature in psychosocially stressed male tree shrews. Moreover, chronic clomipramine treatment reversed symptoms of depression, such as weight loss, anhedonia, fluctuations in locomotor activity, sustained urinary cortisol elevation, irregular cortisol rhythms, and deficient hippocampal long-term potentiation (LTP) in subordinate tree shrews (Wang et al., 2013a). These findings all suggest that tree shrews are suitable for studying depression.

### Drug addiction models

Drug addiction is a chronic and relapsing brain disease. Tree shrews are useful animal models for the study of human drug addiction pathogenesis due to their well-developed central nervous system. Opitz & Weischer (1988) observed that non-deprived, unstressed tree shrews preferred nicotine solution over water in a free-choice situation. In addition, Wiens et al. (2008) revealed that wild tree shrews naturally preferred alcohol. Morphine, an effective analgesic, induces tolerance and addiction in animals. Sun et al. (2012) injected tree shrews with morphine for 7 days and induced conditioned place aversion (CPA) along with withdrawal symptoms. Intramuscular injection of morphine (5 or 10 mg/kg) has been shown to significantly increase the locomotor activity of tree shrews, with morphine-conditioned tree shrews exhibiting place preference in the morphine-paired chamber and naloxone-precipitated withdrawal inducing place aversion in chronic morphine-dependent tree shrews (Wang et al., 2012a). Cocaine and amphetamine regulated transcript peptides are also reported to show similar expression patterns in tree shrews and primates (Ábrahám et al., 2005). These findings suggest that tree shrews are suitable for studying human drug addiction.

## NERVE RELATED DISEASES

### Myopia models

Chickens and monkeys are the most commonly used animal models for myopia study; however, tree shrews might be better candidates due to their low cost, short experiment cycle, small size, and similar eye development to humans. Axial myopia has been reliably produced in tree shrews raised with eyelid closure (Marsh-Tootle & Norton, 1989) and following the application of agents to block collagen crosslinking (McBrien & Norton, 1994). Dark treatment has also resulted in a shift toward myopia in tree shrews (Norton et al., 2006). Tree shrews demonstrated significant scleral thinning and tissue loss, particularly at the posterior pole of the eye, after monocular deprivation of pattern vision (McBrien et al., 2001). The scleral gene expression levels are regulated by the visual environment during the development of myopia and recovery (Guo et al., 2014; He et al., 2014; Siegwart & Norton, 2002). The use of positive lenses has been shown to inhibit myopia in tree shrews, suggesting that daily intermittent positive lens wear might effectively prevent myopia progression in children (McBrien et al., 2012; Siegwart & Norton, 2010). Taken together, tree shrews appear to be ideal animal models for studying myopia.

### Alzheimer's disease (AD) models

Alzheimer's disease is one of the most common neurodegenerative diseases. The central pathological feature of AD is the profuse deposition of amyloid-β protein (Aβ) in the brain parenchyma and vessel walls. The sequence of the tree shrew Aβ protein has very high homology (92% identity) with the human Aβ protein (Pawlik et al., 1999). Fan et al. (2013) revealed that genes related to AD in tree shrews share a high degree of homology with human beings. Yamashita et al. (2012) observed senile plaque-like structures in the frontal cortex and nucleus accumbens of aged tree shrews. He et al. (2013) induced AD in tree shrews by intracerebroventricular injection of Aβ. Lin et al. (2016) injected Aβ1-40 into the tree shrew hippocampus and induced cognitive lesions associated with neuronal apoptosis. Thus, tree shrews are a potentially effective animal for studying AD.

### Transgenic tree shrews

Transgenesis is an important technology for the establishment of disease models. To date, site-directed gene editing has been applied to generate transgenic animals (Capecchi, 2005). Additionally, the development of CRISPR/Cas9 technology has made transgenesis more extensively applicable (Mali et al., 2013). Li et al. (2017) found a culture system for the long-term expansion of tree shrew spermatogonial stem cells without the loss of stem cell properties and successfully generated transgenic offspring by transplanting enhanced green ﬂuorescent protein (EGFP) into sterilized adult male tree shrew testes. Such research has greatly consolidated the position of the tree shrew in the field of disease animal models.

## CONCLUSIONS AND PERSPECTIVES

The tree shrew is more closely related to humans than to rodents in evolution and offers distinct advantages in breeding (Fan et al., 2013). This species has garnered increasing attention in China and worldwide in terms of its use as a novel animal model for a variety of human diseases. To date, tree shrews have shown unique advantages in the study of hepatitis, depression, drug addiction, and myopia. The successful application of transgenic techniques in tree shrews (Li et al., 2017) will broaden the choices for researchers in selecting appropriate animal models for studying human diseases.

However, several hurdles still exist in this field. To date, there are no tree shrew strains with a pure genetic background. Scientists at KIZ, CAS have worked very hard to establish tree shrew strains in recent years, but have only obtained fourth generation offspring by inbreeding so far. Additionally, gene knockout techniques have yet to be applied in tree shrews. Moreover, physiological research tools and materials for the tree shrew remain relatively underdeveloped. This includes tree shrew specific antibodies, although most antibodies against human proteins are suitable for detecting tree shrew proteins. Current tree shrew disease models need to be optimized and studied in depth, and, as such, we to need exhibit patience before adopting the tree shrew in the study of all human diseases. There is, however, no doubt that the tree shrew will play an increasingly important role in the biomedical field.

**1:** Tree shrew disease models

Disease model		Methods	Characteristics	References
	HBV	Infected with 0.1 mL of serum from HBV positive patients by intramuscular injection	More than half of the tree shrews showed anorexia and emaciation 5 days after infection. Both HBsAg-and HBsAb-positive tree shrews reached 9/22 at 15-35 days after infection.	Pang et al., 1981
		Neonatal tree shrews infected with 0.3 mL of HBV inoculum by two subcutaneous injections	13% of neonatal tree shrews showed long-term (more than 48 weeks) chronic infection with HBV. Hepatic histopathological changes were observed in chronically HBV-infected animals.	Wang et al., 2012a
		Infected with human HBV serum, then fed AFB1 diluted with milk, 150 μmg/kg, 6 days/week for 105 weeks	HCCs developed in 120.3±16.6 weeks at incidences of 67%	Li et al., 1999b
	HCV	Infected intravenously with 0.15 mL of serum from patients with chronic hepatitis C	34.8% of tree shrews developed HCV viremia at different times during 47 weeks of follow-up. Peaks in transaminases, high ALT levels, and anti-HCV antibodies were observed	Xie et al., 1998
	HDV/HBV	Infected with HBV-RNA and HDV-RNA serum (0.2 mL) by tail vein injection	HBsAg-and HDAg-positive serum was detected in 6/13 tree shrews 4-5 weeks after inoculation with high ALT levels.	Li et al., 1995
	HEV	Infected with 0.2 mL of swine genotype 4 HEV by intravenous injection	HEV RNA was detected in the feces, liver, spleen, kidneys, and bile of tree shrews. Histological examination showed that HEV caused acute liver lesions. Infected tree shrews showed positive IgG and IgM antibodies.	Yu et al., 2016
	H1N1	Infected with ~10^5^ H1N1 by intranasal administration	3/3 of the infected tree shrew displayed mild systemic and respiratory symptoms and pathological changes in respiratory tracts 14 days after infection.	Yang et al., 2013
	HSV	Infected with 10^6^ PFU of HSV-1 McKrae virus inoculum on each eye without ocular scarification	About 10% of the tree shrews showed severe nervous system disease symptoms, such as ataxia, astasia, torticollis, star gazing, and other abnormal behaviors from 5 days post infection	Li et al., 2016a
	Bacteria	Infected with 5×10^6^ CFU of *Staphylococcus* *aureus* on the surface of a wound	*S. aureus* caused persistent infection for 7 days and inflammatory response for 4 days after inoculation.	Li et al., 2012
		Infected with 2×10^6^ CFU of *Pseudomonas* *aeruginosa* on the surface of a wound	Dacron graft infection model caused persistent infection for 6 days, with pus observed 3 days after inoculation	
Cancer	Hepatocellular carcinoma (HCC)	Administration of highly purified aflatoxin B1 intermittently in the diet at 2 mg/ kg	9/12 tree shrews developed HCCs between 74 and 172 weeks. Liver tumors in all nine tree shrews were well to poorly differentiated.	Reddy et al., 1976
		Infected with human HBV serum and aflatoxin B1 (200-400 μg/kg body weight per day) for 6 days every week, totally 15-16 mg	For 83-137 weeks, the incidence of HCC was significantly higher in tree shrews infected with HBV and exposed to AFB1 (52.94%) than in those solely infected with HBV (11.11%) or exposed to AFB1 (12.50%). All 13 cases of liver tumor were HCC, including eight cases of trabecular type, three of adenoid type, and two poorly-differentiated.	Yan et al., 1996
	Breast cancer	Spontaneous breast papillary tumor	Tumor cells were positive for PR, highly proliferative, and less apoptotic compared with normal breast epithelial cells.	Xia et al., 2012
		Oral administration of 20 mg of DMBA once every 3 weeks, three times in total in 30 tree shrews, with15 tree shrews implanted with 150 mg of MPA	After 25-33 weeks, tumor incidence in the DMBA plus MPA group reached 50%. All DMBA plus MPA-induced tumors were positive for PR and ERα but negative for HER2. The *PTEN/ PIK3CA*, but not *TP53 and GATA3*, genes were frequently mutated in the breast tumors.	Xia et al., 2014
		Overexpression of the *PyMT* oncogene by lentivirus (10 μL) nipple injection	Most tree shrews developed mammary tumors with a latency of about 3 weeks, and by 7 weeks all injected tree shrews had developed mammary tumors. *PyMT*-induced tree shrew mammary tumors were predominately papillary carcinomas.	Ge et al., 2016
	Lung cancer	DHPN was administered at a dose of 250 mg/kg body weight subcutaneous once a week for 80 weeks	Between 65 and 102 weeks, 78%-89% of tree shrews developed pulmonary adenomas. Clara cells were the main components of these tumors. In two DHPN-treated males, bronchioalveolar carcinomas were observed and 9% of the DHPN-treated animals developed squamous cell carcinomas of the skin and HCC.	Rao & Reddy, 1980
Metabolic diseases	Diabetes	STZ (60, 70, 80 mg/kg) was intraperitoneally injected twice on the first and third day	After 9-16 weeks, the success rates for the 60, 70, and 80 mg/kg STZ injection groups were 66.7%, 66.7%, and 100%, respectively. Tree shrews displayed increased fasting blood and urine glucose, impaired oral glucose tolerance, and disturbed lipids metabolism and renal function.	Wu et al., 2013
	Fatty liver	Treated with alcohol solutions (10% and 20%) for two weeks	After 14 days, the serum ALT, AST, GGT, TC, and TG levels of the alcohol-treated groups significantly increased. Animals exhibited obvious pathological changes, including swelling of the hepatocytes and disarrangement of cell cords.	Xing et al., 2015
		High fat, cholesterol, and cholate diet (HFHC, 20% fat, 1.25% cholesterol and 0.5% sodium cholate by weight)	After 10 weeks, HFHC caused blood dyslipidemia, and induced hepatic lipid accumulation and liver inﬂammation. HFHC also caused liver fibrosis.	Zhang et al., 2015
	Blood Stasis	Intraperitoneal injection of 25, 50, and 75 mg/kg doses of carrageen glue for 3 days	One day after treatment, tree shrews were in low spirits. Tongue vein was enlarged. Regular probability pain increased and the colors of the tongue, claw, and naso-labial area became darker with increasing dose.	Chen et al., 2016
	Thrombotic cerebral ischemia	The scalp was incised to expose the right skull, which was irradiated with a 560-nm filtered beam for 10 min	Sodium, calcium, and water contents increased to a maximum after 4 hours in ischemic penumbra.	Li et al., 1999a
Mental diseases	Depression	Two male tree shrews were housed in a pair-cage, 1 h direct conflict (fighting) and 23 h indirect influence for 21 days	After 21 days, the subordinate tree shrews showed alterations in body weight, locomotion, avoidance behavior, and urinary cortisol levels.	Wang et al., 2011
	Drug addiction	Intramuscular injection of morphine at increasing doses (5, 10, 15, 20 mg/kg body weight for 7 days). Naloxone (1.25 mg/kg body weight) induced CPA	After 7 days, the tree shrews developed morphine tolerance and chronic morphine dependence with increasing doses.	Sun et al., 2012
		Nicotine solution (10 mg/L nicotine tartrate) in drinking water	Tree shrews preferred nicotine solution, with this drug-taking behavior stable over 14 months.	Opitz & Weischer, 1988
Nerve related diseases	Myopia	Placement in continuous darkness for 10 days	After 10 days, the dark-treatment group eyes shifted toward myopia, and the vitreous chamber became elongated relative to normal eyes	Norton et al., 2006
		Monocular deprivation of pattern vision for short-term (12 days) or long-term (3-20 months) periods	Significant scleral thinning and tissue loss, particularly at the posterior pole of the eye, were associated with ocular enlargement and myopia development after both short-and long-term treatments	McBrien et al., 2001
PFU: plaque forming units; CFU: colony forming unit.				
